# Two Cases of Natural Infection of Dengue-2 Virus in Bats in the Colombian Caribbean

**DOI:** 10.3390/tropicalmed6010035

**Published:** 2021-03-12

**Authors:** Alfonso Calderón, Camilo Guzmán, Teresa Oviedo-Socarras, Salim Mattar, Virginia Rodríguez, Víctor Castañeda, Luiz Tadeu Moraes Figueiredo

**Affiliations:** 1 Faculty of Veterinary Medicine and Animal Production Husbandry, Institute for Biological Research in the Tropics (IIBT), University of Cordoba, Monteria 230002, Cordoba, Colombia; acalderonr@correo.unicordoba.edu.co; 2 Department of Pharmacy, Faculty of Health Sciences, Institute for Biological Research in the Tropics (IIBT), University of Cordoba, Monteria 230002, Cordoba, Colombia; cguzman40@hotmail.com; 3 Research Group on Tropical Animal Production (GIPAT), Faculty of Veterinary Medicine and Animal Production Husbandry, University of Cordoba, Monteria 230002, Cordoba, Colombia; toviedo@correo.unicordoba.edu.co; 4 Bacteriological Program, Microbiological and Biomedical Research Group of Cordoba (GIMBIC), Faculty of Health Sciences, University of Cordoba, Monteria 230002, Cordoba, Colombia; vrodriguez@correo.unicordoba.edu.co; 5 Veterinary Diagnostic Laboratories Network, Colombian Agricultural Institute, Cerete 230550, Cordoba, Colombia; jose.castaneda@ica.gov.co; 6 Center for Virological Research, Faculty of Medicine, University of Sao Paulo, Riberao Preto 05508-060, Brazil; ltmfigue@fmrp.usp.br

**Keywords:** antibodies, arbovirus, flavivirus, immunohistochemistry, pathology, zoonoses

## Abstract

Dengue, a mosquito-borne zoonotic disease, is the most common vector-borne disease in tropical and subtropical areas. In this study, we aim to demonstrate biological evidence of dengue virus infection in bats. A cross-sectional study was carried out in the departments of Cordoba and Sucre, Colombia. A total of 286 bats were captured following the ethical protocols of animal experimentation. The specimens were identified and euthanized using a pharmacological treatment with atropine, acepromazine and sodium pentobarbital. Duplicate samples of brain, heart, lung, spleen, liver, and kidney were collected with one set stored in Trizol and the other stored in 10% buffered formalin for histopathological and immunohistochemical analysis using polyclonal antibodies. Brain samples from lactating mice with an intracranial inoculation of DENV-2 were used as a positive control. As a negative control, lactating mouse brains without inoculation and bats brains negative for RT-PCR were included. Tissue sections from each specimen of bat without conjugate were used as staining control. In a specimen of *Carollia perspicillata* captured in Ayapel (Cordoba) and *Phylostomus discolor* captured in San Carlos (Cordoba), dengue virus was detected, and sequences were matched to DENV serotype 2. In bats RT-PCR positive for dengue, lesions compatible with viral infections, and the presence of antigens in tissues were observed. Molecular findings, pathological lesions, and detection of antigens in tissues could demonstrate viral DENV-2 replication and may correspond to natural infection in bats. Additional studies are needed to elucidate the exact role of these species in dengue epidemics.

## 1. Introduction

Dengue is a zoonotic disease transmitted by arbovirus, endemic in the tropics, where all the environmental conditions for its circulation are found [1]. In Latin America, the number of cases increased; the last reported outbreak in 2019 produced 3,140,872 cases [2]. In February 2020, the incidence rate of dengue was 81.51 cases per 100,000 population, and this forced the Pan American Health Organization (PAHO) to release an epidemiological alert for dengue in the Americas. The incidence of dengue in Colombia was 255 per 100,000 individuals [3].

Emerging and re-emerging infectious diseases have become one of the most severe threats to public health. Approximately 75% of the diseases that have emerged during the last two decades have wildlife as their source [4,5]. Dengue virus (DENV) is an enveloped, icosahedral flavivirus. The genome is a single unsegmented linear chain of ribonucleic acid (RNA) of positive polarity that varies between 10.5 and 11 Kilobase [6]. Signs and symptoms of DENV infections in humans vary from a nonspecific febrile syndrome to fatal encephalitis and hemorrhagic fever. DENV is highly prevalent in tropical countries. Along with malaria, it is the most significant disease transmitted by vectors [7,8].

Dengue is commonly diagnosed by detection of antibodies, antigens, and nucleic acids detection using reverse transcriptase polymerase chain reaction (RT-PCR) [9]. In exceptional cases, it is also detected by immunohistochemistry (IHC), which identifies antigens in tissues [10]. Using IHC, DENV antigens have been detected in live patients’ biopsy samples and post-mortem specimens via pathology techniques [11].

Bats provide environmental services through control of insects, dispersal of seeds, and pollination [12] and have a wide global distribution [13]. Although bats can harbor many infectious viruses [14] they do not develop apparent disease signs from any virus. It is believed that the increase in body temperature caused by flight increases metabolic rate and mitochondrial activity. This triggers an immune response that includes interleukins and prostaglandins production, which may prevent the establishment of viral diseases [15,16,17]. The present work aims to demonstrate natural infection of DENV in bats from two departments in the Caribbean region of Colombia.

## 2. Materials and Methods

### 2.1. Sampling, Geographic Area, and Capture of Specimens

In 2017, we conducted a descriptive, cross-sectional study using convenience sampling. A total of 12 geographic zones were selected, eight in Cordoba and four in Sucre. This included the main ecosystems of these two departments in the Colombia Caribbean. Cordoba and Sucre’s departments were chosen because they are endemic for human DENV infection [8,18]. The Ethics Committee of the Faculty of Veterinary Medicine and Zootechnics at the University of Cordoba, Colombia approved this study. To capture the bats, we followed the rules of research with non-commercial animals from the National Environmental Authority of Colombia. For 12 nights, we used five mist nets (each 6 m× 2.5 m), which were left open from 18:00 to 22:00 h. Pregnant or lactating females were released at the capture site. Bats were identified with taxonomic keys based on morphometric parameters [19] and were initially sedated with an intramuscular injection of atropine (0.11 mg/kg) after which they were euthanized with an intracardiac injection of sodium pentobarbital (0.2 mL). The euthanizing was applied by overdose using 0.2 mL of sodium pentobarbital by intracardiac. Brain, heart, lung, liver, kidney, and spleen samples were extracted at the site of capture. Tissues were stored in cryovials with Trizol (Invitrogen, Carlsbad, CA, USA) and in liquid nitrogen for molecular studies. Another fraction was stored in 10% formalin to perform histopathology and immunohistochemistry techniques.

### 2.2. Molecular Methods

**Figure 1:**
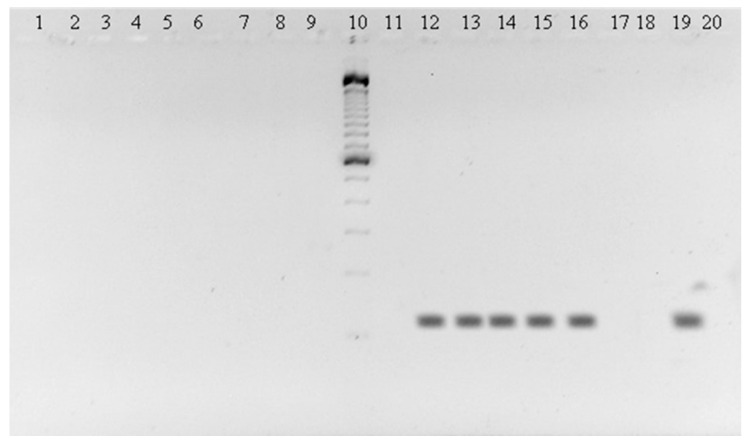
143 bp amplicons of *C. perspicillata* in brain, (lane 2), heart (lane 13), lung (lane 14), liver (lane 15), spleen (lane 16); YFV positive control (lane 19) and negative control (lane 20). Molecular weight marker 100 pb Invitrogen (lane 10).

Tissues of brain, heart, lung, spleen, liver, and kidney of 286 captured bats from Cordoba and Sucre departments were analyzed. The RNA was extracted with Trizol and cDNA was synthesized using a reverse a reverse transcriptase enzyme M-MLV (Invitrogen), a cDNA was obtained. Nested RT-PCR was performed with a first-round to get an amplicon product of 1360 bp using the primers: Flavi 1+(5′-GAYYTIGGITGYGGIGIGGIRGITGG-3′) and Flavi 1-(5′-TCCCAICCIGCIRTRTCRTCIGC-3′), and Flavi 2+(5′-GYRTIYAYAWCAYSAT GGG-3′) and Flavi 2-(5′-CCARTGITCYKYRTTIAIRAA ICC-3′) for a second-round to obtain an amplicon product of 143 bp. Degenerated primers were designed based on conserved from a region of gene *NS5*, which encodes for the polymerase, to align with known flaviviruses sequences [20]. As a control for each species, complementary primers were used to sequence a mitochondrial gene mt DNA from bats [21]. The Yellow Fever Virus (YFV) vaccine prepared with an attenuated live virus strain 17D-204 (Sanofi-Pasteur, Lyon, France) was used as a positive control. As a negative control, molecular water grade was used. The obtained amplicons (Figure 1) were sequenced in both forward and reverse directions by Sanger method at Macrogen (Korea)

The obtained amplicons (Figure 1) were sequenced by the Sanger method at Macrogen (Korea).

### 2.3. Phylogenetic Analysis

This analysis involved sequences of the four DENV, including the two sequences detected in bats from Cordoba. The records were downloaded from GenBank and are displayed in the tree. Thirty-six sequences were aligned using Clustal, the model was Hasegawa-Kishino-Yano (HKY), with 1000 bootstrap and the phylogenetic reconstruction was done with Maximum Likelihood method (ML). All procedures were performed with MEGA X software [22].

### 2.4. Histopathology

The tissues were dehydrated with increasing concentrations of isopropanol and xylol and placed in liquid paraffin to form blocks. Four uM thick slices of tissues were cut and stained with hematoxylin-eosin (Merck KGaA, Darmstadt, Germany) and covered with a coverslip and Entelan (Spectrum Chemical, New Brunswick, NJ, USA). Pathologic lesions were read and interpreted using a camera microscope (Leica-DM500, Leica-Microsystems, Wetzlar, Germany). PCR-negative specimens of the same species were included.

### 2.5. Immunohistochemistry

Four-micron histological sections were placed on ColorFrost Plus slides (Thermo Scientific, Waltham, MA, USA) at 58 °C for two hours. Antigenic recovery was performed under pressure (Cuisinart Pressure Cooker Model CPC-600) with Trilogy™ (Cell Marque, Rocklin, CA, USA) at 1:100 dilutions for 15 min at 125 °C. Endogenous peroxidase was blocked with 9% H_2_O_2_ diluted in methanol for 15 min. The sections were delineated with Dakopen (SDL, Des Plaines, IL, USA), and the tissues were covered with the antibody diluted 1:100 with anti-dengue 1 + 2+3 + 4 (ab26837, Abcam, Cambridge, UK) for one hour. HiDef Amplifier (Cell Marque) was added for 10 min at room temperature. HiDef HRP Polymer Detector (Cell Marque) was added for 10 min at room temperature. The tissue was covered with the Chromogen Liquid DAB + Substrate Chromogen System (Dako North America, Carpinteria, CA, USA) and stained with hematoxylin for one minute. As a negative control, the anti-dengue antibody was replaced with 1% phosphate-buffered saline (PBS). As positive controls, brain samples from suckling mice with an intracranial inoculation of DENV-2 were used. PCR-negative specimens of the same species were included.

## 3. Results

During 12 nights of sampling, 23 species belonging to six families were caught. Table 1 shows the number of species per group food sources.

**Figure 2:**
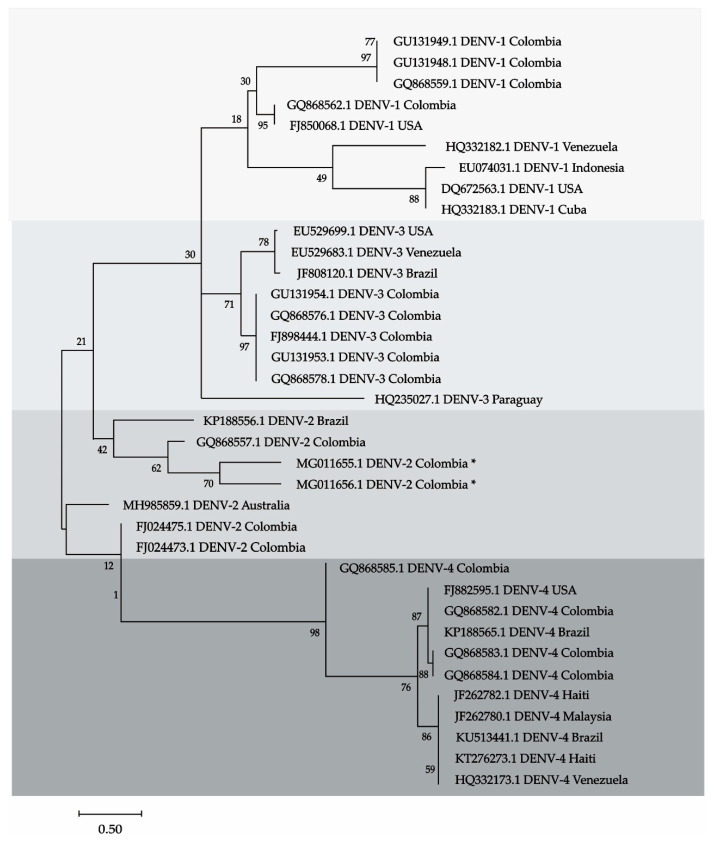
Phylogenetic tree showing 4 clades that correspond to the 4 serotypes of the DENV. Sequences MG011655 and MG011656 (marked with an asterisk) were detected in bats from Cordoba, Colombia, the sequences are in the clade, these sequences correspond to serotype 2 of DENV. DENV-2.

Dengue was detected in two bats, one *Carollia perspicillata* and one *Phylostomus discolor* from Ayapel and San Carlos (Cordoba). The sequences matched to DENV-2. Amplicons in the brain, heart, lung, liver, and spleen of *C. perspicillata* are shown (Figure 1). The sequences of the amplicons were deposited in the GenBank, *C. perspicillata* (CIIBT-106-2) with accession number MG011655 and *P. discolor* (CIIBT-1932) with the accession number MG011656 [23] (Figure 2).

**Figure 3:**
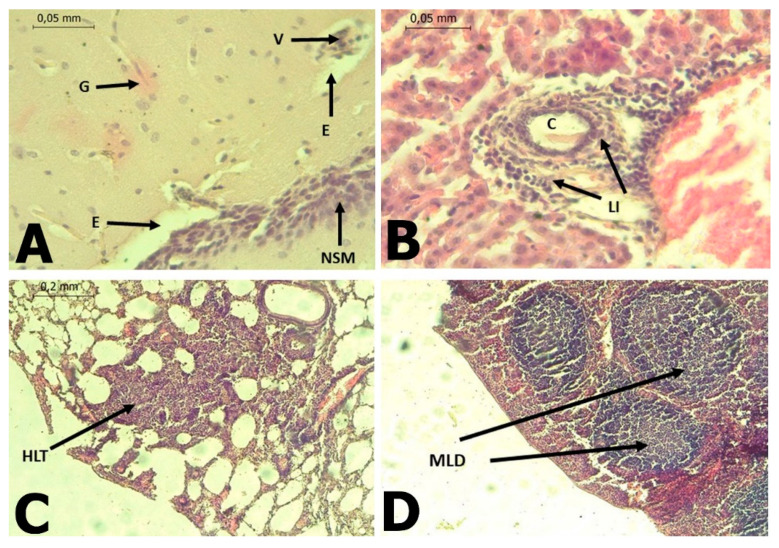
(**A**): Brain of *C. perspicillata* with the presence of gliosis (**G**), perineural edema (**E**), vasculitis (**V**), non-suppurative meningitis (NSM) of lymphoid type B. (H&E stain 400×). (**B**): Liver of *C. perspicillata* with lymphoid infiltrate (LI) around portal triad (C) (H&E stain 400×). (**C**): Lung of *C. perspicillata* with hyperplasia of lymphoid tissue associated with bronchi (HLT), thickening of alveolar septa, interstitial pneumonia (H&E stain 100×). (**D**): Spleen of *C. perspicillata* with moderate lymphoid depletion (MLD) in lymph node (H&E stain 100×).

**Figure 4:**
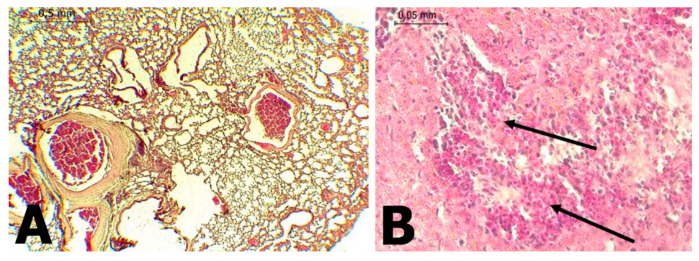
(**A**): Lung of *P. discolor* with presence of congestion (H&E stain 100×). (**B**): Liver of *P. discolor* with severe eosinophilic lymphocyte mixed necrotic hepatitis and (H&E stain 400×).

**Figure 5:**
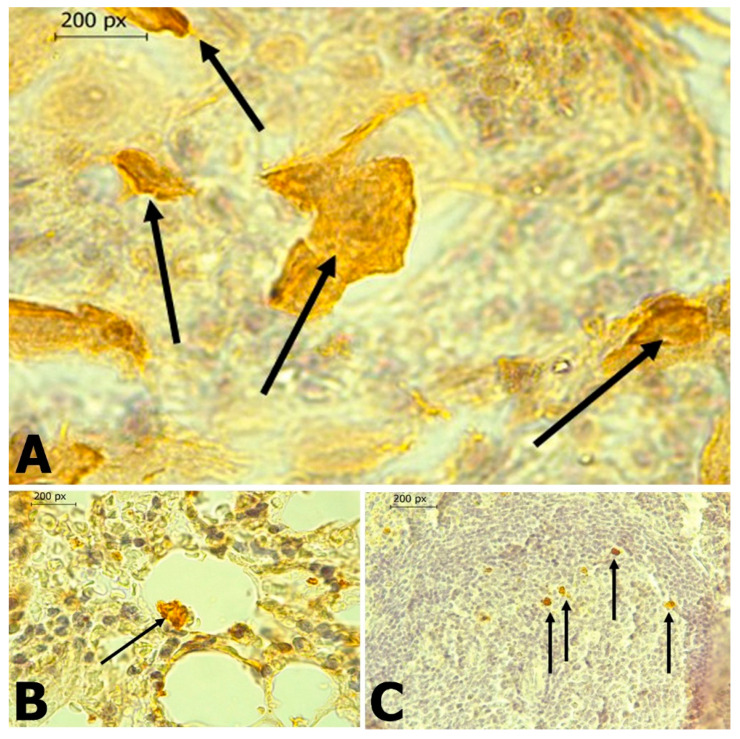
(**A**): Brain of *C. perspicillata* which shows the presence of antigens for DENV using IHC. (400×). (**B**): Lung of *C. perspicillata* which shows the presence of antigens for DENV using IHC. (400×). (**C**): Spleen of *C. perspicillata* which shows the presence of antigens for DENV using IHC. (400×).

**Figure 6:**
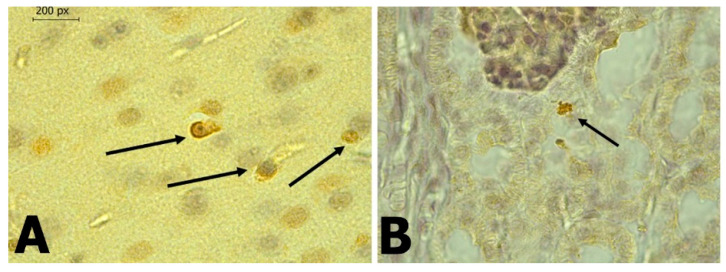
(**A**): Brain of *P. discolor* which shows the presence of antigens for DENV using IHC. (400×). (**B**): Kidney of *P. discolor* which shows the presence of antigens for DENV using IHC. (400×).

**Figure 7:**
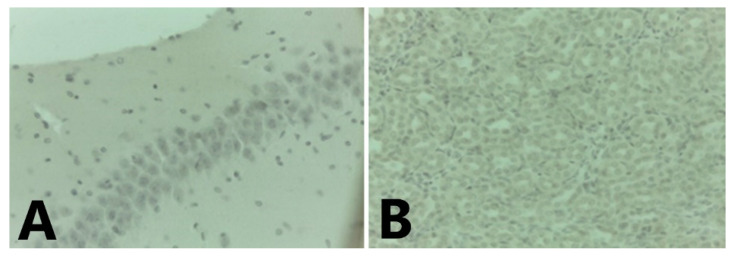
Staining controls: Mouse brain (**A**) without infection (400×). (**B**): Bat´s kidney PCR negative to Dengue (400×).

In *C. perspicillata* captured in Ayapel, lesions compatible with viral infection (Figure 3) brain (A), liver (B), lung (C), and spleen (D) were observed. In the *P. discolor*, captured in San Carlos lesions compatible with viral infection (Figure 4) in the lung (A) and liver (B) were observed. DENV virus antigens were found in the (Figure 5) brain (A), lung (B) spleen (C) of *C. perspicillata.* DENV and (Figure 6) in the brain (A) and kidney (B) of *P. discolor*. Staining controls (Figure 7A) in the mouse brain and (Figure 7B) bat´s kidney.

## 4. Discussion

The presence of dengue virus serotype two was detected molecularly in different tissues of two bats, captured in an area of the Colombian Caribbean. Previous studies in Mexico and Ecuador [24], Mexico [25,26,27,28,29], French Guian [30] have demonstrated the presence of DENV by serological and molecular techniques. The phylogenetic tree confirms that the sequences detected MGO11655.1 in *C. perspicillata* and MGO11656.1 in *P. discolor* in the Colombian Caribbean are DENV-2. The sequences were grouped within a clade whin DENV-2 sequences from other countries, with a similarity of 87% and a branch support of 62.

In the infected *C. perspicillata* and *P. discolor* brain, gliosis and immunostaining areas of gliosis restorative, neuronal death with replacement by astrocytes and infiltration of mononuclear cells were observed. These lesions have also been observed in immunocompromised mice infected with DENV-2 [31]. The results for the *C. perspicillata* and *P. discolor* brain are compatible with our histopathological findings. DENV-2 can cause nerve tissue damage due to plasma extravasation, hemorrhage, or immune responses [32,33].

Hyperplasia of lymphoid tissue associated with bronchi was observed, along with the thickening of the alveolar septa in *C. perspicillata* and pulmonary congestion in *P. discolor*. In fatal dengue cases in humans, the alveolar septa’s thickening has also been observed [34,35]. The presence of mononuclear infiltrates [34,36], edema, diffuse alveolar hemorrhage [35,36], congestion [36], and interstitial pneumonia, suggests that at least in some situations, viruses may be the primary cause [37]. It is known that DENV viral replication occurs in macrophages and leukocyte cells [36].

The *C. perspicillata* and *P. discolor* lungs’ are similar to the histopathological changes described in DENV infections in humans [34,35,36,37]. Nevertheless, studies on mice intraperitoneally inoculated with DENV-1 (dengue virus serotype 1) did not show inflammatory cells. This could be because of the route of inoculation or, lack of an animal model that adequately mimics human disease and provokes a similar adaptive immune response. However, viral RNA was detected [38,39]. Neuronal necrosis is the main structural change in the infection of the central nervous system [40] and apoptosis has been associated with the accumulation of viral proteins [41].

Lymphoid-type mononuclear infiltration was found around the portal triad in the liver of *C. perspicillata*. Additionally, severe hepatic necrosis with mixed infiltration was observed in the portal triad, along with vacuolar changes, in the liver of *P. discolor*. In human dengue cases, the liver is one of the commonly affected organs [42] because hepatocytes and Kupffer cells are target cells for viral replication [43,44]. After internalization, apoptotic damage related to tumor necrosis factor is induced [37,45]. Additionally, liver damage result from direct viral toxicity or the immunopathological effect of the response to DENV [42]. DENV infection in hepatocytes induces the formation of autophagosomes, which favor viral replication [46]. Acute liver damage is variable in DENV infections and has not been associated with any degree of viremia or extravasation [47]. Fatty changes have been observed in hepatocytes [42], but they may be a consequence of metabolic disorders in infected livers [38].

The pathological findings and the presence of DENV antigens in brain, lung, and spleen samples, suggest viral replication of dengue virus in the bat species; however, they do not demonstrate viral replication of DENV in two *C. perspicillata* and *P. discolor* bats. This is possible due to the presence of DENV receptors such as glycoproteins, heparin sulfate, mannose, and phosphatidylserine in mammalian cells, that facilitate the penetration of DENV by endocytosis [42,48]. These mechanisms enable the spread of DENV to skin, liver, spleen, lymph nodes, kidneys, bone marrow, lungs, thymus, and brain [49,50]

No lesions were observed in the kidney of *P. discolor*. However, DENV antigens were detected with IHC. Acute kidney injury is a complication of dengue, and the cytopathic effect is directly caused by viral antigens attached to glomerular structures [50,51]. In infected human kidneys, viral antigens were detected in tubular epithelial cells using IHC and in situ hybridization [36,52]. This is also seen with yellow fever in tubular cells, where antigens of the virus can be found [53].

In a study of kidney cells from rats infected with DENV-1, no viral RNA was detected. This suggests that viral replication did not occur in the renal tissue. In our study, the absence of viral RNA in the renal tubular cells suggests no viral replication [50] even though immune complexes could reabsorb the antigens after elimination through the kidney. This is also seen with yellow fever in tubular cells, where antigens of the virus can be found [53].

In the DENV-2, PCR-positive *C. perspicillata* specimen, the spleen’s lymphatic nodules presented moderate lymphoid depletion, and DENV antigens were found using IHC. The presence of viral antigens in the spleen could be a consequence of a high concentration of mononuclear cells, which allow for the replication of DENV [31,54,55].

In Brazil, DENV infections were reported in larvae of *A. albopictus* (transovarial transmission) and *Haemagogus leucocelaenus*, suggest a possible sylvatic cycle [56]. Recently in Mexico, DENV was found in Diptera order Streblidae (*S. Wiedemann* and *T. parasiticus parasiticus*) that parasitized bats [29]. Mosquitos genera *Aedes, Culex, Anopheles, Culiseta, Mansonia, Coquillettidia, Psorophora, Armigeres, Myzorhynchus* and *Taeniothyncus* have been reported in Cordoba, Colombia [57,58]. DENV-2 has been found in *A. aegypti,* Yellow Fever Virus, St. Louis encephalitis virus, and West Nile virus in female blood-sucking mosquitoes [58,59].

Regarding the presence of DENV in bat tissues, experimental studies have shown low viral titers by RT-PCR and seroconversion rates in *Artibeus spp, Artibeus intermedius* and great fruit-eating bats (*A. jamaicensis*), which indicates that these species are not an adequate reservoir for the virus [27,60,61]. On the other hand, experimental infections in yinpterochiroptera (*Pteropus giganteus*) did not evidence clinical signs of disease. Meanwhile, in yangochiroptera (*Myotus lucifugus*) resulted in clinical sign development of the disease [62,63]. Nonetheless, the search for natural infection in bats from Mexico and French Guyana detected nucleic acids from DENV in blood and liver [30,61]. Additionally, anti-DENV antibodies have been detected in bats from Uganda [64]. A study carried in bats from Costa Rica detected the virus by RT-PCR and serology but did not isolate it [28]. These results suggest that bats are not an adequate reservoir for DENV and possibly, infection in bats may be due to human-to-bat spillover [65].

The limitations of the present work were that it was not possible to isolate the virus by culture, probably because the viral titers in the tissues were very low. Likewise, it was not possible to have complete genome sequences using NGS techniques. Therefore, the sequences detected in the present study are short. Additionally, blood samples for serological assays were not collected. However, the study provides a methodology for the surveillance of emerging and re-emerging viruses in wild species that could be hosts of pathogens of importance in public health.

## 5. Conclusions

Molecular detection and sequencing of DENV-2 in bats, the presence of pathological lesions compatible with a viral infection, and detection of polyclonal antigens for DENV in C. perspicillata and *P. discolor*, could be the result of natural infection of DENV in bats of the Colombian Caribbean, and they could be accidental reservoirs. Additional studies are needed to elucidate the exact role of these species in dengue epidemics.

## Figures and Tables

**Table 1:** Distribution of bats species by food sources.

Food Source	Captured Species	No	Food Source	Captured Species	No
**Insectivorous**	*Phyllostomus discolor*	42	**Frugivorous**	*Artibeus planirostris*	99
	*Molossus molossus*	14		*Carollia perspicillata*	38
	*Saccopteryx bilineata*	4		*Artibeus lituratus*	30
	*Eptesicus brasiliensis*	1		*Sturnira lilium*	20
	*Rhogeessa yo*	2		*Carollia brevicauda*	1
	*Eumops glaucinus*	1		*Carollia castanea*	1
	*Lasiurus ega*	1		*Uroderma bilobatum*	11
	*Micronycteris microtis*	1	**Piscivorous**	*Noctilio albiventris*	3
	*Myotis nigricans*	1		*Noctilio leporinus*	3
	*Saccopteryx leptura*	1	**Nectarivorous**	*Glossophaga soricina*	6
	*Molosops temminckii*	1	**Hematophagous**	*Desmodus rotundus*	4
**Omnivorous**	*Trachops cirrhosus*	1			
**Subtotal**		**70**			**216**
**Total**	**286**				

## Data Availability

Not applicable.
